# Clonal Analysis of the T-Cell Response to *In Vivo* Expressed *Mycobacterium tuberculosis* Protein Rv2034, Using a CD154 Expression Based T-Cell Cloning Method

**DOI:** 10.1371/journal.pone.0099203

**Published:** 2014-06-06

**Authors:** Susanna Commandeur, Mariateresa Coppola, Karin Dijkman, Annemieke H. Friggen, Krista E. van Meijgaarden, Susan J. F. van den Eeden, Louis Wilson, Jolien J. van der Ploeg-van Schip, Kees L. M. C. Franken, Annemieke Geluk, Tom H. M. Ottenhoff

**Affiliations:** Department of Infectious Diseases, Leiden University Medical Center, Leiden, The Netherlands; University of Cape Town, South Africa

## Abstract

Tuberculosis (TB), caused by *Mycobacterium tuberculosis* (*Mtb*), remains a leading cause of death worldwide. A better understanding of the role of CD4^+^ and CD8^+^ T cells, which are both important to TB protection, is essential to unravel the mechanisms of protection and to identify the key antigens seen by these T cells. We have recently identified a set of *in vivo* expressed *Mtb* genes (IVE-TB) which is expressed during *in vivo* pulmonary infection in mice, and shown that their encoded antigens are potently recognized by polyclonal T cells from tuberculin skin test-positive, *in vitro* ESAT-6/CFP10-responsive individuals. Here we have cloned T cells specific for one of these newly identified *in vivo* expressed *Mtb* (IVE-TB) antigens, Rv2034. T cells were enriched based on the expression of CD154 (CD40L), which represents a new method for selecting antigen-specific (low frequency) T cells independent of their specific function. An Rv2034-specific CD4^+^ T-cell clone expressed the Th1 markers T-bet, IFN-γ, TNF-α, IL-2 and the cytotoxicity related markers granzyme B and CD107a as measured by flow cytometry. The clone specifically recognized Rv2034 protein, Rv2034 peptide p81–100 and *Mtb* lysate. Remarkably, while the recognition of the dominant p81–100 epitope was HLA-DR restricted, the T-cell clone also recognized a neighboring epitope (p88–107) in an HLA-DR- as well as HLA-DQ1-restricted fashion. Importantly, the T-cell clone was able to inhibit *Mtb* outgrowth from infected monocytes significantly. The characterization of the polyfunctional and *Mtb* inhibitory T-cell response to IVE-TB Rv2034 at the clonal level provides detailed further insights into the potential of IVE-TB antigens as new vaccine candidate antigens in TB. Our new approach allowed the identification of T-cell subsets that likely play a significant role in controlling *Mtb* infection, and can be applied to the analysis of T-cell responses in patient populations.

## Introduction

Tuberculosis (TB), caused by *Mycobacterium tuberculosis* (*Mtb*), accounts for ≥1.5 million deaths each year and remains one of the leading causes of death due to infectious disease. There is no efficient vaccine against TB yet, since the only *Mtb* vaccine available, *Mycobacterium bovis* bacille Calmette-Guerin (BCG), induces limited and variable protection against pulmonary TB, the transmissible form of the disease. Novel *Mtb* vaccines, including improved BCG strains, attenuated *Mtb* strains and subunit vaccines, are currently under study [1]. Given the major role of CD4^+^ and CD8^+^ T cells during infection with *Mtb*, better understanding of the role and function of these T cells during *Mtb* infection and post (BCG) vaccination is of key importance to developing further improved vaccines [2].

Advanced flow cytometry allows for detailed characterization of specific T-cell subsets. Due to major advances in the development of improved instruments and reagents an increasing number of parameters can be measured simultaneously [3], [4]. The measurement of IFN-γ production is generally used to detect *Mtb* antigen-specific T cells. However, this approach is inherently biased towards detecting Th1 cells, and potentially falls short of detecting other antigen specific T-cell subsets. Although in theory the use of T-cell activation markers, such as CD25 and CD69 can circumvent such bias, substantial levels of CD25 and CD69 expression are detected also in unstimulated samples, indicating that their induction is not strictly antigen induced [5], [6]. Expression analysis of CD154 (CD40L) may provide a suitable alternative to overcome these issues. CD154 has previously been described as an antigen-specific induced cell surface marker, which is transiently expressed on T cells upon specific antigen recognition via TCR [6], [7]. CD154 interacts with CD40, which leads to subsequent activation of APC, driving both humoral and cellular immunity [8], [9].

Recently we identified a set of novel *in vivo* expressed *Mtb* (IVE-TB) antigens as possible TB vaccine candidate antigens [10]. One of the IVE-TB encoding genes, *Rv2034,* was found to be expressed during inflammatory pulmonary infection, and its encoded protein was strongly recognized by T cells from mycobacteria exposed individuals [10]. Moreover, vaccination of HLA-transgenic mice with Rv2034 protein significantly reduced *Mtb* load (Commandeur *et al.* unpublished data). Understanding the immunological response to IVE-TB antigens, as well as to early-phase expressed (*e.g.* ESAT-6) or late phase-expressed (*e.g.* DosR regulon and Rpf) proteins [11], [12] is important to the development of better TB vaccines and TB biomarkers. Therefore we performed a more detailed analysis of the T-cell response to *Mtb* IVE-TB antigen Rv2034 using an Rv2034 specific CD4^+^ T-cell clone that was generated using a novel CD154-expression based cell selection method described here, and analyzed both its specificity and phenotype. The T-cell clone was specific for an HLA-DR restricted epitope in Rv2034 p81–100. Surprisingly, this was neighbored (p88–107) by a both HLA-DR and HLA-DQ presentable epitope, and in agreements with this the p81–107 sequence was found to possess multiple promiscuous MHC class II binding features. Furthermore, the T-cell clone recognized *Mtb* lysate, and expressed the Th1 markers T-bet, IFN-γ, TNF-α and IL-2 and cytotoxic markers granzyme B and CD107a, consistent with a Th1 phenotype that co-expresses cytotoxicity granule markers. Importantly, the T-cell clone was able to inhibit *Mtb* outgrowth from infected monocytes.

## Materials and Methods

### Recombinant Proteins

Recombinant (fusion) proteins were produced as previously described [13]. In short, gene amplified PCR products were cloned by Gateway Technology (Invitrogen, San Diego, CA, USA) in a bacterial expression vector containing an N-terminal hexa-histidine (His) tag. Generated vectors were sequenced to confirm correct insertion of the product. Recombinant proteins were overexpressed in *Escherichia coli* strain BL21 (DE3) and further purified. The size and purity of the proteins were analyzed by gel electrophoresis, using Coomassie brilliant blue and Western blotting using an anti-His antibody (Invitrogen, Carlsbad, CA, USA). Endotoxin contents were below 50 EU (endotoxin unit) per mg of recombinant protein as tested using a Limulus Amebocyte Lysate (LAL) QCL-1000 assay (LonzaInc., Basel, Switzerland). Recombinant proteins were tested to exclude protein non-specific T-cell stimulation and cellular toxicity in IFN-γ release assays using PBMC of *in vitro* PPD-negative, healthy Dutch donors recruited at the Blood Bank Sanquin, Leiden, The Netherlands [14], [15]. None of these controls had experienced any known prior contact with TB patients.

### Study Subjects

Blood samples were collected by venipuncture. Peripheral blood mononuclear cells (PBMCs) were isolated from heparinized venous blood by Ficoll density gradient centrifugation and stored in liquid nitrogen until further use. HLA typing was performed at the Leiden University Medical Center, Department of Immunohematology and Blood Transfusion, The Netherlands, under supervision of dr. Dave Roelen and prof. Frans Claas. For Sequence Based Typing (allele level, 4 digit) of HLA-A, -B, and -C, the sequences of exons 2 and 3 were determined. Gene-specific amplification and sequencing primers were developed to sequence each exon separately. Primers were located in introns or adjacent exons to enable complete sequencing of exons 2 and 3. To distinguish the alleles A*2402/*2409N/A*2411N additional sequencing of exon 4 was needed. To distinguish Cw*1701/02/03, Cw*07011/012/06, and Cw*1801/02 the sequence of exons 1 or 5 was necessary in addition.

### Ethical Statement

Donors gave written informed consent before blood donation. The study protocol (P207/99) was approved by the Institutional Review Board of the Leiden University Medical Center.

### Synthetic Peptides

Rv2034 20-mer peptides with 10 aa overlap were synthesized by Peptide 2.0 Inc. (Chantilly, VA, USA) [16]. Generated peptides were analyzed by HPLC to determine purity of peptide products (>75%). Mass-spectrometry was performed to determine the molecular weight of the peptides.

### Enzymatic Digestion of *Mtb* Lysate

*Mtb* lysate was generated as previously described [10]. To generate hypoxic *Mtb* lysate, H37Rv was grown to late log phase under a flow of nitrogen containing oxygen tension for 24 hours in a shaking incubator at 37°C [17]. To generate starvation *Mtb* lysate, H37Rv was grown to log phase, washed and incubated for an additional 7 days in PBS at 37°C [18]. *Mtb* lysates were treated with Proteinase K (Promega, Madison, WI, USA) and Lysozyme (Sigma Aldrich, St. Louis, MO, USA), both at a final concentration of 5 µg/ml for 1 hour at 37°C in a shaking incubator. Subsequently, the enzymes were inactivated for 90 minutes at 99°C.

### Lymphocyte Stimulation Test

PBMC (1.5*10∧^5^) were cultured in triplicates in 96-well round-bottom plates (Nunc, Roskilde, Denmark) and incubated with or without protein (10 µg/ml) in a final volume of 200 µl AIM-V medium (Invitrogen, Breda, Netherlands) at 37°C and 5% CO_2_. After 6 days, supernatants were harvested and used for IFN-γ determination. Subsequently, cells were pulsed with [^3^H]-thymidine (0.5 µCi/well) and incubated 16–18 hours at 37°C and 5% CO_2_. Incorporation of [^3^H]-thymidine was measured using a Wallac 1450 MicroBetaTriLux Liquid Scintillation Counter (Wallac, Turku, Finland).

### Generation of CD154 Specific T-cell Clones

PBMC (0.5–1*10∧^6^) were seeded in 96-wells round-bottom plates together with peptide pool mix (1 µg/ml per peptide) in Iscove's Modified Dulbecco's Medium (IMDM; Gibco, Invitrogen, Breda, the Netherlands) containing 10% pooled human serum (HuS) and human IL-2 (Proleukin; Novartis Pharmaceuticals UK Ltd., Horsham, UK) at a final concentration of 100 IU/ml and incubated for 1 week at 37°C and 5% CO_2_. After 1 week, the cells were restimulated with peptide pool (1 µg/ml per peptide) and autologous PBMC (0.5–1*10^∧6^) in IMDM containing 10% HuS and 1∶100 diluted anti-CD40 antibody (clone HB14, MiltenyiBiotec, Auburn, CA, USA) and incubated 16 hours at 37°C and 5% CO_2_. CD154 positive cells were sorted and seeded in 96-wells round-bottom plates (0.3, 1 and multiple cell(s)/well) containing IMDM, irradiated (3000 rad) feeders (5*10∧^4^ cells/well), IL-2 (100 IU/ml), 10% HuS and phytohemagglutinin (PHA) at a final dilution of 4 µg/ml (Remel, Oxoid, Haarlem, the Netherlands). After one week IL-2 was added (100 IU/ml) and cultured for approximately another 1–2 weeks until clonal expansion was established.

### Magnetic-Activated Cell Sorting (MACS) of CD154 Positive Cells

Stimulated PBMC were collected and labeled with anti-CD154 phycoerythrin (PE) (MiltenyiBiotec, Auburn, CA, USA). Subsequently cells were washed and incubated with anti-PE MicroBeads (MiltenyiBiotec, Auburn, CA, USA). Labeled and washed cells were applied to an MS Column (MiltenyiBiotec, Auburn, CA, USA) to enrich for CD154 positive T cells. Samples collected prior cell sort, CD154-enriched population and flow through were additionally stained for anti-CD3 Pacific Blue (PB) (Biolegend), anti-CD4-Alexa Fluor 700 (eBioscience), anti-CD8 HorizonV500 (BD Biosciences) (Figure S1). MACS buffer (PBS supplemented with 2mM EDTA and 2% FCS) was used during cell sorting procedure.

### RNA Isolation and cDNA Synthesis

RNA was isolated from 5*10∧^6^ T cells using 1 ml Trizol Reagent (Invitrogen, Life Technologies, Paisley UK) according to the manufacturer’s protocol and dissolved in 40 µl RNAse free H_2_O (Ambion, Invitrogen, Life Technologies, Paisley, UK). RNA concentration was determined by measuring the OD_260_ with the Nanodrop ND1000 (Thermo Scientific, Waltham, MA). Complementary first strand DNA was synthesized from 0.6 µg RNA, and with 10 pmol oligoDt primer (Life Technologies, Paisley, UK). This mixture was first incubated at 72°C for 2 minutes to facilitate primer annealing. Superscript III Reverse Transcriptase (1 µl), 10 nmol dNTPs, 0.1 mM DTT (all Invitrogen, Life Technologies, Paisley, UK) were added to this mixture and subsequently incubated at 42°C for 60 minutes. Amplification was performed by adding 2 µl of cDNA, 10 nmol dNTPs, 20 pmol oligoDt primer, 20 pmol capswitch primer and 38 µl H_2_0 in a total volume of 50 µl. The amplification PCR program consisted of an initial denaturation cycle of 1 minute at 95°C, followed by 20 amplification cycles (5 seconds at 95°C, 5 seconds at 65°C and 6 minutes at 68°C). The quality of the amplified cDNA was analyzed by gel electrophoresis.

### Determination of T-cell Clonality

Clonality of the clone was assessed by PCR amplification of the Vβ and Vα regions, with a constant primer [19] for the Cα and Cβ region and 26 and 37 V region-specific primers for the Vβ and Vα region, respectively [19]. Mastermix contained 3 (Vβ) or 4 (Vα) µl cDNA, 5 pmol reverse primer, 200 µM dNTPs, 1.5 mM MgCl_2_, 0.5 unit of GoTaq Flexi DNA polymerase and 1x Green GoTaq Flexi Polymerase Buffer (all Promega, Madison, WI) and 1x bovine serum albumin (BSA, New England Biolabs, Ipswich, MA). 5 pmol of each V-specific primer was added. A total volume of 10 µl was used for PCR amplification. An initial denaturation step at 95°C for 2 minutes was followed by 35 cycles of denaturation at 94°C for 30 seconds, annealing at 65°C (Vα) or 60°C (Vβ) for 30 seconds and extension at 72°C for 30 seconds. Finally, a 5 minute extension step was performed at 72°C. PCR products were analyzed by standard gel electrophoresis. Sequencing templates were amplified using Phusion DNA Polymerase (New England Biolabs, Ipswich, MA) and a constant primer extended with an M13 sequence (TGTAAAACGACGGCCAGT). Amplicons spanning the variable, CDR3 and joining regions were purified using Wizard SV Gel and PCR clean up system (Promega, Madison, WI) according to manufacturers’ protocol. Samples were sequenced at Baseclear (www.baseclear.com) using a separate M13 primer.

### Restimulation of T-cell Clones

Irradiated (3000 rad) feeders were pulsed with peptide mix (20 µg/ml per peptide) for 2 hours and washed. Clones were seeded in 96 wells at 2*10∧^4^/well together with the feeders (1*10∧^5^/well). For CD4^+^ T cells, clones were incubated in IMDM containing 10% HuS and 1% LeucoA (Sigma Aldrich, St. Louis, MO, USA) at 37°C and 5% CO_2_. After three days, IL-2 was added (25 IU/ml). For CD8^+^ T cells, clones were incubated in IMDM contain 10% HuS, IL-7 and IL-15 both at a final concentration of 5 ng/ml (PeproTech Inc., Rocky Hill, NJ, USA). After three days IL-2 (50–100 IU/ml) was added.

### T-cell Subset Analysis Using Flow Cytometry

Feeders (3000 rad) were seeded to 96 flat- bottom plate (1*10∧^5^) and incubated for 4–6 hours. The plates were washed three times with RPMI. T cells were added, together with antigen (10 µg/ml), peptide or peptide pool (5 µg/ml per peptide). After 2–4 hours 3 µg/ml Brefeldin A (BFA; Sigma Aldrich, St. Louis, MO, USA) was added and incubated overnight (o/n). Cells were harvested and collected in 5 ml round-bottom FACS tubes (BD Biosciences), washed and stained for T-helper markers. For additional flow cytometry, irradiated PBMC (2000 rad) were seeded together with antigen (10 µg/ml) or peptide (5 µg/ml) to 96 flat-bottom plate (1*10∧^5^) and incubated o/n. Plates were washed with RPMI and T cells were added (1*10∧^5^). After 2–4 hours 3 µg/ml Brefeldin A (BFA; Sigma Aldrich, St. Louis, MO, USA) was added and incubated overnight (o/n). Cells were harvested and collected in FACS tubes, washed and stained.

Cells were stained with LIVE/DEAD fixable violet dead cell stain (ViViD; Invitrogen, Carlsbad, CA, USA) prior surface and intracellular staining to discriminate between live and dead cells according to manufacturer’s instructions. CD14 and CD19 markers were included to eliminate monocytes and B cells using a DUMP channel [20]. Following ViViD, surface markers were stained in PBS 0.1% BSA for 1/2–1 hour at 4°C. Subsequently intracellular staining (ICS) was performed using fixation and permeabilization (Intrastain) kit (DakoCytomation, Glostrup, Denmark). Unstimulated and unstained samples were included as negative controls. Different T-cell subset panels were used. All included, *surface staining*; anti-CD3 PE-Cy5 or anti-CD3 PE-Cy7, (both BD Biosciences), anti-CD4 Texas Red (Caltag), anti-CD8 HorizonV500 (BD Biosciences), anti-CD14 PB (Invitrogen) and anti-CD19 PB (Invitrogen), *ICS;* anti-CD154 APC-Alexa eFluor 780 (eBioscience). In addition, the following markers were used for (i) **T-helper subset panel**, *surface staining*; anti-TCRα/β Fluorescein isothiocyanate (FITC), *ICS*; anti-IFN-γ Alexa Fluor 700 (BD Pharmingen), anti-TNF-α PE-Cy7 (BD Biosciences) and anti-IL-2 PE (BD Pharmingen) (anti-Granzyme B APC (Caltag)). (ii) **Transcription factor subset panel**, *ICS*; anti-IFN-γ Alexa Fluor 700 (BD Pharmingen), anti-T-bet eFluor660 (eBioscience), anti-GATA-3PerCP-eFluor 710 (eBioscience), anti-Ror-γt PE (eBioscience) and anti-IL-17 FITC (eBioscience). (iii) **Regulatory T-cell subset panel**, *surface staining*; anti-CD25 PE-Cy5 (BD Biosciences) and anti-CD39 PE (Biolegend), *ICS*; anti-Foxp3 Alexa Fluor 700 (eBioscience) and anti-IL-10 APC (MiltenyiBiotec). (iv) **T-cell cytotoxicity subset panel**, *ICS*; anti-Granzyme B APC (Caltag), anti-IFN-γ Alexa 700 (BD Pharmingen), anti-CD107a PE-Cy5 (BD Pharmingen) and anti-Perforin PE (BD Pharmingen). Of note, anti-CD107a PE-Cy5 was already added during incubation of the T cells with APC and antigen. Fluorescence minus one (FMO) controls were included for each T-cell subset panel. Data were acquired on a BD LSRFortessa (BD Biosciences) and analyzed using Flowjo version 7.6.5 (Tree Star Inc., Ashland, OR, USA).

### Antigen Specificity Test

T cells (1*10∧^4^) were seeded in 96 flat-bottom plates in presence of HLA class I and/or class II (mis)matched irradiated (2000 rad) PBMC (5*10∧^4^) [21]. T cells were stimulated with either protein (10 µg/ml), peptide (10 µg/ml), PHA (2 µg/ml) or purified protein derivative (PPD; Statens Serum Institute, Copenhagen, Denmark; 5 µg/ml) and incubated for 3 days at 37°C and 5% CO_2_. At day 3, supernatant was harvested and tested for IFN-γ production. Subsequently, cells were pulsed with [^3^H]-thymidine and incubated 16–18 hours at 37°C and 5% CO_2_. Incorporation of [^3^H]-thymidine was measured using a Wallac 1450 MicroBetaTriLux Liquid Scintillation Counter.

### IFN-γ ELISA

IFN-γ concentrations in triplicate, pooled supernatants were measured using a standard ELISA technique according to the manufacturer’s instructions (U-CyTech, Utrecht, The Netherlands). Samples were tested in duplicate. Concentrations were calculated with Microplate manager software (MPM) using a standard concentration curve included in the assay. The cut-off value for positivity was set arbitrarily at 100 pg/ml, as in our previous studies.

### Blocking of HLA-DR and HLA-DQ Molecules

Irradiated (2000 rad) PBMC (5*10∧^4^) and monoclonal antibodies (20 µg/ml) directed against HLA-DR (B8.11.2) or HLA-DQ (SPV-L3) molecules (provided by Prof. F. Claas, Department of Immunohematology and Blood Transfusion, Leiden University Medical Center, Leiden, The Netherlands) were seeded in 96 flat-bottom plates and incubated for 2 hours. T cells (1*10∧^4^) and protein antigen, peptide (5 µg/ml) or PHA (2 µg/ml) were added and co-incubated for 3 days at 37°C and 5% CO_2._ Both IFN-γ production and proliferation were determined as previously described. Rp15_1-1_ was included as reference CD4^+^ T-cell clone [22], [23].

### Western Blot Analysis

Recombinant protein (0.4 µg), *Mtb* lysates (10 µg) and ultralow molecular weight marker (Color Marker Ultra Low Range; Sigma Aldrich, St. Louis, MO, USA) were separated under denaturizing conditions using a 17% Tricine sodium dodecyl sulfate-polyacrylamide gel (SDS-PAGE) at 100–200 V for 2–3 hours [24]. Proteins were transferred onto a polyvinylidenedifluoride (PVDF) membrane by electroblotting (40V; 250 mA). The membranes were blocked using 5% skim milk (Fluka, Biochemika, Sigma Aldrich, 70166), washed (PBS 0.1% Tween20) and incubated with Rv2034-immunized or naive mouse serum. After incubation blots were washed and incubated with secondary horseradish peroxidase (HRP)–conjugated rabbit anti-mouse Ig, detecting total IgG, IgA and IgM (P0260 Dako, Glostrup, Denmark). Protein bands were visualized using chemiluminescence (ECL) HRP substrate (Amersham ECL Select, GE Healthcare Bio-Sciences AB, Uppsala, Sweden). Blots were developed using Fuji super RX medical X-ray films.

### Generation of Monocyte Derived DC

PBMC were thawed and monocytes were isolated by positive selection using MACS CD14 MicroBeads (MiltenyiBiotec, Auburn, CA, USA) following manufacturer’s instructions. Monocytes were seeded in T75 flasks (Corning) in RPMI medium containing 10% heat-inactivated fetal calf serum (FCS, 50 U/ml penicillin, 50 µg/ml streptomycin (Gibco, Paisley, United Kingdom), IL-4 and GM-CSF (Biosource International, Camarillo, CA, USA) both at 10 ng/ml for 6 days to generate monocyte-derived immature DCs. Mature DCs were generated by stimulating DCs with 10 ng/ml lipopolysaccharide (LPS) for an additional 24 hours. DC differentiation and maturation was validated by flow cytometry using anti-CD14 PE (BD Pharmingen), anti-HLA-DR FITC (BD Pharmingen), anti-CD1a Alexa Fluor 700 (Biolegend), anti-CD163 Alexa Fluor 647 (Biolegend), anti-CD80 PE-Cy7 (Biolegend), anti-CD86 PE-Cy5 (BD Pharmingen) and anti-CD3 PB (Biolegend).

Matured DCs were loaded with Rv2034 protein, Rv2034 p81–100 and different conditions of *Mtb* lysate and incubated for 24 hours. Cells were washed and T-cell clone added. After 2 hours BFA was added and culture incubated o/n. Activation of T cells was determined by detection of CD154 and Th1 markers using the T-helper subset panel.

### *Mtb* Inhibition Assay

Autologous PBMC were isolated using Ficoll density centrifugation from venous, heparinized blood and plated in quadruplicate cultures (1*10∧^6^ cells/well; assuming 10% monocytes) in 48-wells plates (Costar Corporation, Cambridge, Mass.) in PBS. After overnight incubation at 37°C, PBS was removed and replaced by IMDM containing 10% human serum of 37°C. The adhering monocytes were infected with *Mtb* H37Rv at an MOI of 10. After overnight incubation at 37°C, T-cell clone was added at effector:target (E:T) ratios of 20∶1 and 50∶1. After an additional o/n incubation, cells were lysed. Lysates were homogenized in PBS and the number of bacteria was determined by culturing serial dilutions of the homogenates on 7H10 agar plates (BD Biosciences) supplemented with BD BBL Middlebrook OADC enrichment (100 ml per bottle; BD Biosciences). Colonies were counted after 2–3 weeks incubation at 37°C.

### Statistical Analysis

GraphPad Prism software was used for statistical analysis. A Mann- Whitney *U* test was used to analyze the difference in CFU. A *p-*value≤0.05 was considered significant.

## Results and Discussion

### Generation of T-cell Clones for Subsequent Phenotypic and Functional Analysis

We previously reported that *in vivo* expressed *Mtb* (IVE-TB) antigens have significant vaccine potential [10] [Commandeur *et al.* unpublished data]. It is thus important to characterize the underlying immune responses to these antigens in more detail, particularly in humans. To this end, clonally expanded antigen specific T cells are powerful tools [25], [26]. To generate IVE-TB specific T cells we used a T-cell cloning method based on CD154 expression to enrich for antigen-specific T cells irrespective of function. We selected two *Mtb* antigens; the well-studied TB10.4 protein [27] and the recently described *in vivo* expressed *Mtb* (IVE-TB) antigen Rv2034 [10].

**Figure 1:**
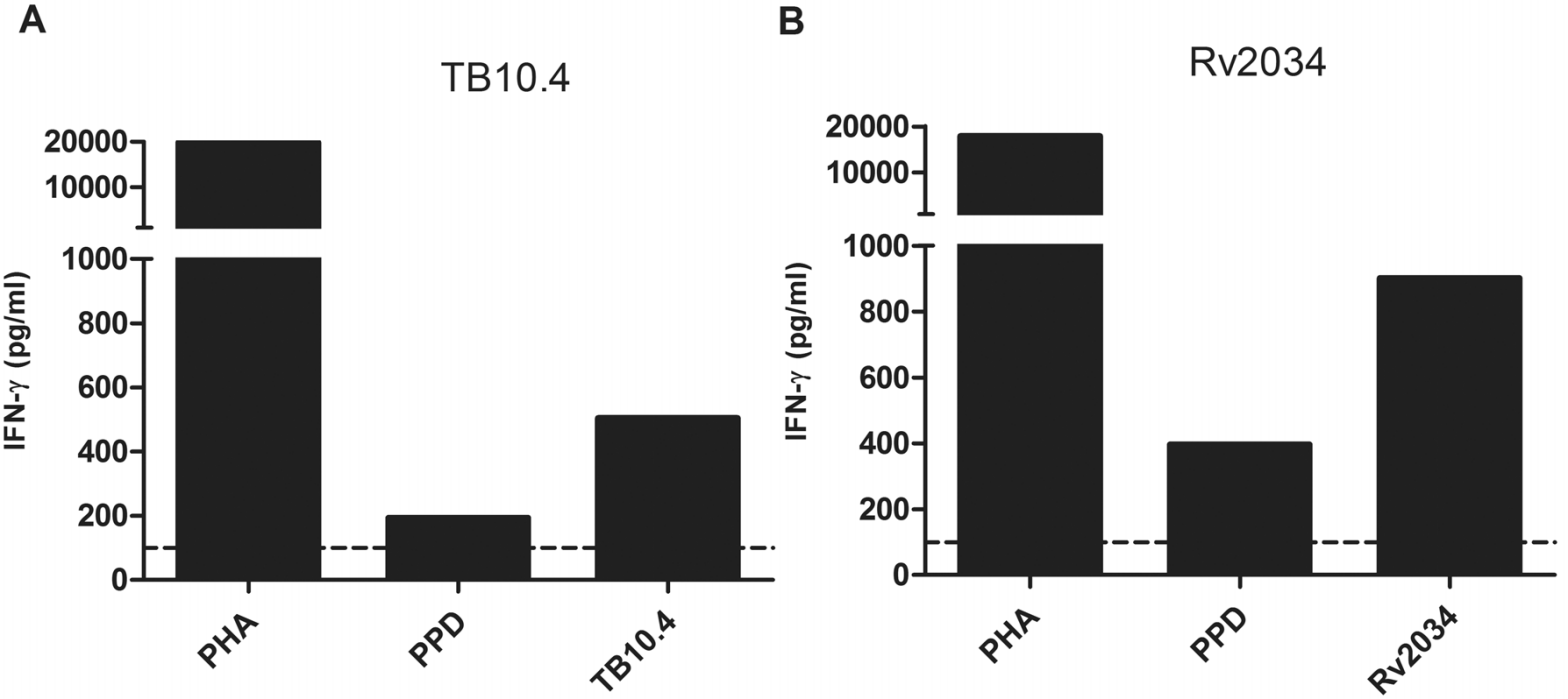
PBMC recognition of TB10.4 and IVE-TB antigen Rv2034. PBMC from a PPD^+^ donor were stimulated with different stimuli for 6 days and IFN-γ production (pg/ml) was determined in the supernatants. Both TB10.4 protein (10 µg/ml) (A) and Rv2034 protein (10 µg/ml) (B) were analyzed as well as control mitogen PHA and *Mtb* derived PPD (A and B). Medium values (unstimulated PBMC) were subtracted. IFN-γ concentrations were determined from triplicate-pooled supernatant. A cut-off value was set arbitrarily at 100 pg/ml.

First, PBMC of an *in vitro* PPD^+^ donor known to respond to these antigens (Figure 1A and 1B) were stimulated with peptide pools from TB10.4 or Rv2034 for one week in the presence of IL-2, followed by re-stimulation with the peptide pool for 16 hours, in the presence of anti-CD40 antibodies to inhibit CD154-CD40 interactions [6], preventing loss of surface expressed CD154 [28], [29]. CD154 positive cells were then sorted using Magnetic-activated Cell Sorting (MACS) and seeded at 0.3 or 1 cell per well densities as described in detail in the materials and methods section. To analyze the antigen specificities of the expanded T-cell clones, cell populations were gated based on live CD14^−^CD19^−^CD3^+^ T cells. For TB10.4, eight clones showed responses towards TB10.4 peptide pool stimulation (Table 1). Out of these, five T-cell clones were CD154^+^CD4^+^ and produced either IFN-γ, TNF-α and IL-2, or TNF-α and IL-2. Two clones appeared to be CD8^+^ T cells that produced IFN-γ, with one of these producing a relatively low amount of IL-2 as well. CD154 is known to be expressed on activated CD4^+^ T cells, but can also be upregulated on a fraction of CD8^+^ T cells [30]. However, none of the CD8^+^ T cells expressed CD154. Somewhat surprisingly, one T-cell clone was CD3^+/^CD4^−/^CD8^−^, (double negative (DN) T cells) and did not express CD154 either, nevertheless, CD3^+^ CD4^−/^CD8^−^ are able to express CD154 [31]. This DN T-cell clone produced IFN-γ in response to TB10.4 antigen. The remaining expanded T-cell cultures were TB10.4 non-responsive and are indicated in Table 1.

**Table 1:** Analysis of TB10.4 clonal cultures.

Clone	Subset^a^	CD154	IFN-γ	TNF-α	IL-2
1	**CD4**	+	−	+	+
2	**CD4**	+	+	+	+
3	**DN**	−	+/−	−	−
4	**CD4**	+/−	+/−	+/−	+/−
5	**CD4**	+	+	+	+
6	**CD8**	−	+	−	+/−
7	**CD8**	−	+	−	−
8	**CD4**	+	+/−	+	+

a Single live CD14-CD19-CD3+ cells (population>100 cells).

Non-responding clonal cultures included CD4 (n = 9), CD8 (n = 2) and DN (n = 2) cells.

DN = Double negative.

+ = >1% positive cells.

+/− = <1% positive cells.

For Rv2034, eight antigen responsive T-cell clones were generated using the same approach as described for TB10.4, among which three were CD4^+^ T cells (Table 2). One of these CD4^+^ T-cell cultures showed specific CD154, IFN-γ, TNF-α and IL-2 expression, whereas the remaining two only produced IL-2 in response to antigen. All three identified CD8^+^ T cells produced IFN-γ. As for TB10.4, two potential clones appeared to be DN T cells and also showed a potential to produce IFN-γ and/or IL-2. Proliferating Rv2034 non-responsive T-cell clones are also indicated in Table 2. Thus, using CD154 sorting we were able to generate IVE-TB Rv2034 induced T-cell clones which included CD4^+^, CD8^+^ and DN T cells. Interestingly, besides CD4^+^ and CD8^+^ T cells also DN T cells have been associated with control of intracellular bacteria including *Mtb* [32], [33]. Furthermore, DN T cells also show possible regulatory functions [34], [35]. Besides classically HLA-class Ia restricted cells, the CD8^+^ T-cell clones we isolated might also represent circulating alternative T-cell subsets, such as mucosal associated invariant T (MAIT) cells [36], [37] or HLA-E restricted CD8 T cells [38].

**Table 2:** Analysis of Rv2034 clonal cultures.

Clone	Subset^a^	CD154	IFN-γ	TNF-α	IL-2
1	**DN**	−	−	−	+/−
2	**CD4**	−	−	−	+/−
3	**CD8**	−	+	−	−
4	**CD8**	−	+	−	−
5	**DN**	−	+/−	−	+/−
6	**CD4**	+	+	+	+
7	**CD8**	−	+	−	−
8	**CD4**	−	−	−	+/−

a Single live CD14-CD19-CD3+ cells (population>100 cells).

Non-responding clonal cultures included CD4 (n = 18), CD8 (n = 0) and DN (n = 1) cells.

DN = Double negative.

+ = >1% positive cells.

+/− = <1% positive cells.

### CD4^+^ T-cell Clone Specific for *Mtb* IVE-TB Antigen Rv2034

**Figure 2:**
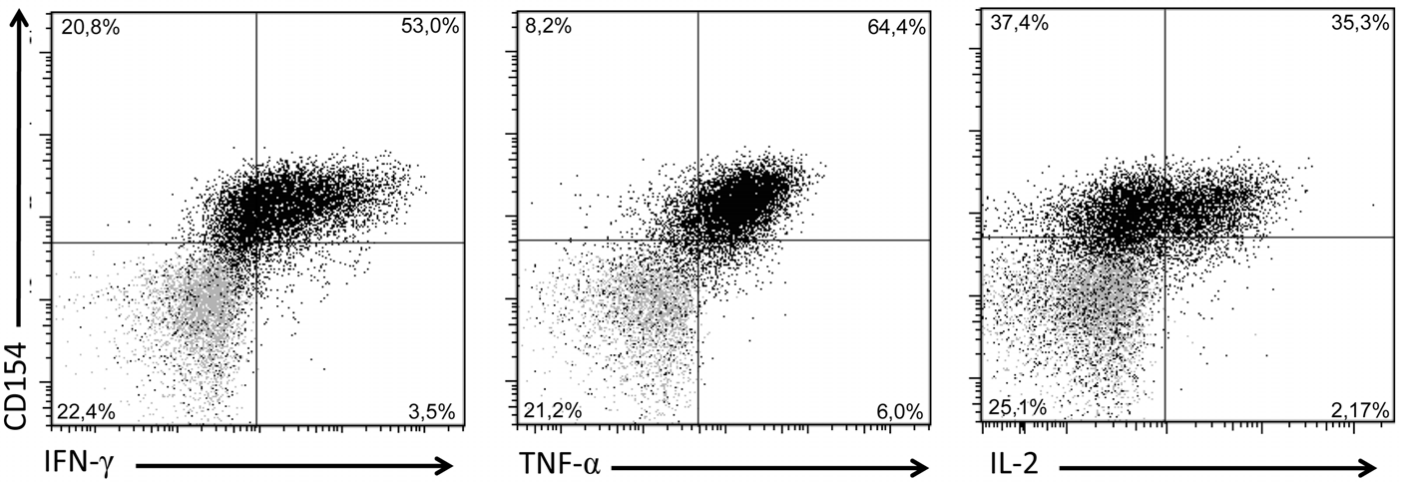
Rv2034 responsive CD4^+^ T-cell clone phenotype. The shown CD4^+^ T-cell clone that had been expanded was restimulated with the Rv2034 peptide pool and analyzed for the expression of CD154 expression, IFN-γ, TNF-α and IL-2 (black dots). Data is representative of over three independent experiments. CD154 and Th1 cytokine expression of non-activated T cells is indicated in grey dots. Dot blots show single live CD14^−^CD19^−^CD3^+^CD4^+^ T cells. The frequency of all CD3^+^CD4^+^ T cell subsets identified upon stimulation are indicated in the corners of each plot.

Thus, both TB10.4 and Rv2034 T-cell clones could be generated using the CD154 sorting method. As the main aim of this work was the analysis of IVE-TB specific T-cell responses, we further studied a CD4^+^ T-cell clone that responded to Rv2034 peptide pool stimulation by CD154, IFN-γ, TNF-α and IL-2 expression (Figure 2). First, its clonality was further confirmed by PCR for both TCRα and TCRβ. The detected variable regions consisted of Vα13, Vα27 and Vβ14, following Arden nomenclature [39] (data not shown). Vα27 was predicted to be an unproductive TCRA rearranged sequence due to an out-of-frame junction, whereas Vα13 was successfully rearranged (the international ImMunoGeneTics database (IMTG)). Thus the clone represents a truly clonal population based on TCR genotyping.

### Identification of T-cell Clone-specific Rv2034 Epitope(s)

**Figure 3:**
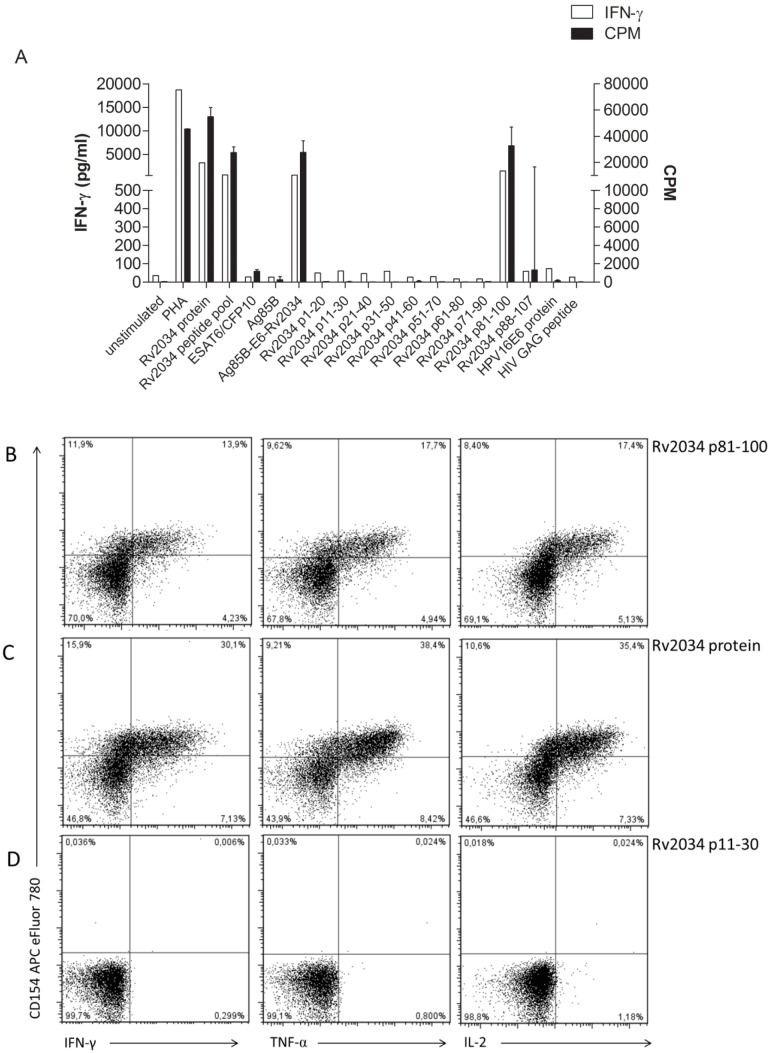
Identification of immunogenic epitope(s) of Rv2034 recognized by CD4 T-cell clone. To identify the immunogenic epitope(s) in Rv2034, the CD4^+^ T-cell clone was stimulated with all individual Rv2034 20-mer peptides with 10 aa overlap; Rv2034 recombinant protein; Rv2034 peptide pool; control ESAT-6/CFP10 fusion protein; an Ag85B/ESAT-6/Rv2034 trimeric fusion protein; and negative and positive control conditions. Autologous irradiated PBMC were used as APCs. Both IFN-γ (open bars) and T-cell proliferation (black bars) were determined. CPM bars represent median ranging the highest and lowest value (n = 3) (A). To determine the Rv2034 p81–100 specific response by flow cytometry, the CD4^+^ T-cell clone was stimulated with Rv2034 p81–100 (B), Rv2034 protein (C) and Rv2034 p11–30 (D) using autologous irradiated PBMC, in the presence of BFA. Intracellular CD154 and Th1 cytokine expression was determined. Data is representative of three independent experiments. Flow cytometry plots show single live CD14^−^CD19^−^CD3^+^CD4^+^ T cells, the frequency of all subsets of CD3^+^CD4^+^ T cells are indicated in the corners of each plot.

To identify the specific T cell epitope(s) of Rv2034, the CD4^+^ T-cell clone was restimulated with single 20-mer peptides from Rv2034. As expected, the Rv2034 peptide pool was strongly recognized by the clone as measured by IFN-γ production and T-cell proliferation (Figure 3A). Rv2034 p81–100 was identified as the dominant immunogenic epitope, while variable responses were observed to Rv2034 p88–107, which overlapped 13 amino acids with Rv2034 p81–100. In addition, recombinant protein Rv2034 and fusion protein Ag85B/ESAT-6/Rv2034 were both recognized, suggesting that Rv2034 epitope is adequately processed from this protein. The fusion protein Ag85B/ESAT-6/Rv2034 includes two early infection phase expressed *Mtb* proteins (Ag85B and ESAT-6; together designated as H1), fused to the *in vivo* expressed Rv2034 protein. Inclusion of multiple infection phase related *Mtb* proteins in a single fusion construct has been shown to improve vaccine efficacy in both mouse and non-human primate models of TB [40], [41], especially if the antigens are expressed during different phases of *Mtb* infection. It is therefore relevant that the immunogenic epitope present in Rv2034 p81–100 is efficiently processed and presented from both the Rv2034 protein and the trimeric Ag85B/ESAT-6/Rv2034 fusion protein. The negative control proteins HPV16E6, Ag85B and ESAT-6/CFP10 fusion protein, and the negative control peptide HIV-GAG were not recognized, in agreement with the strict Rv2034-specificity. As expected, expression of CD154 and Th1 cytokines was detected upon stimulation of the CD4^+^ T-cell clone with Rv2034 p81–100 (Figure 3B) and Rv2034 protein (Figure 3C) whereas no activation was observed upon stimulation with negative control peptide p11–30 of Rv2034 (Figure 3D), further demonstrating the specificity of this CD4^+^ T cell-clone for Rv2034 p81–100.

### T-cell Subset Panel Analysis

**Figure 4:**
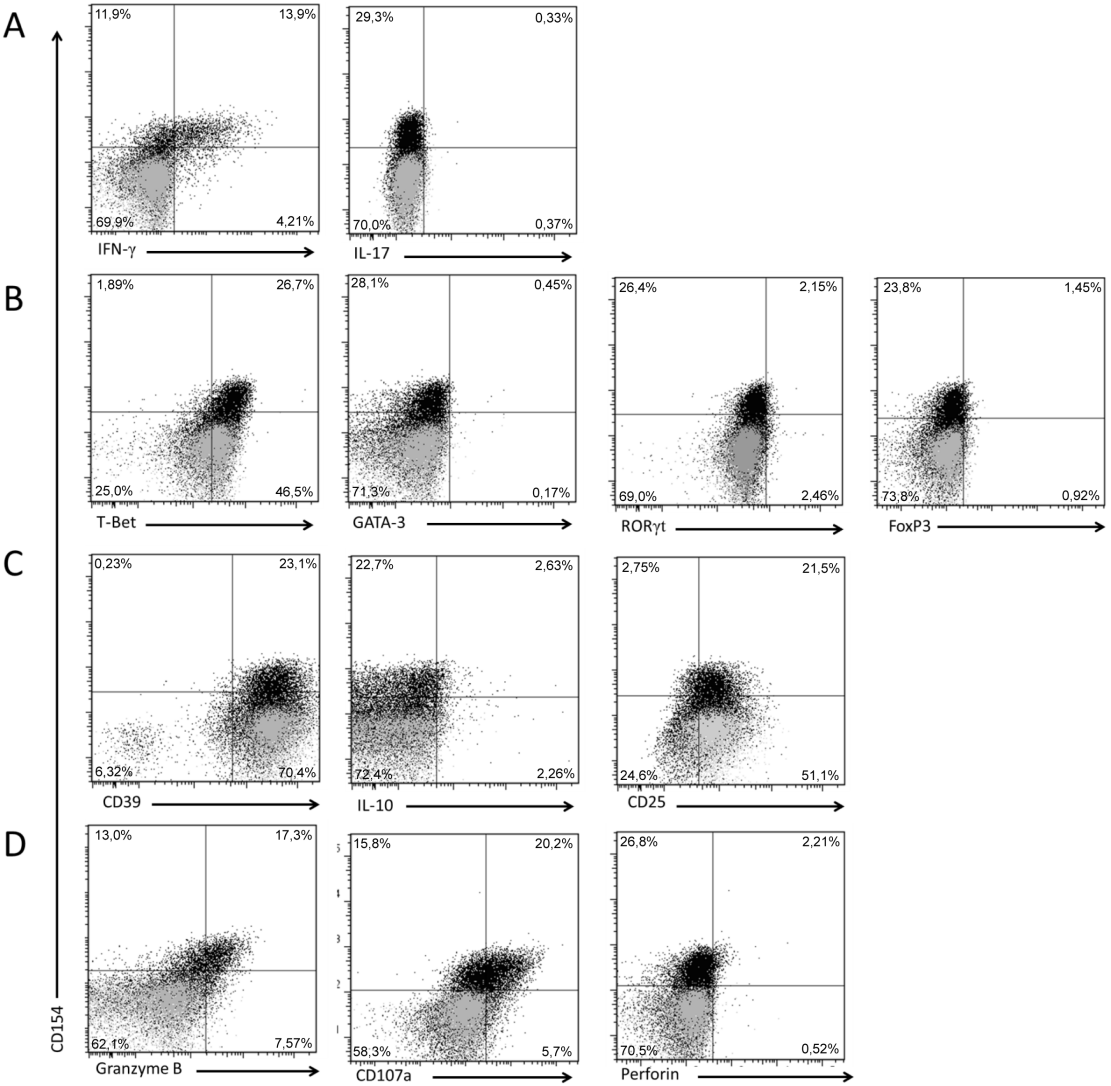
Subset analysis of the CD4 T-cell clone. The CD4^+^ T-cell clone was stimulated with Rv2034 p81–100 in presence of BFA and analyzed the expression of CD154 (A-D), IFN-γ and IL-17 (A) and additional markers: the transcriptional factor markers T-bet (Th1), GATA-3 (Th2), RORγt (Th17) and FoxP3 (Treg) (B); T-cell regulatory markers: CD39, IL-10 and CD25 (C); T-cell cytotoxicity markers: Granzyme B, CD107a and Perforin (D). Markers were analyzed using the mAb subset panels described in the materials and methods section. Data is representative for two independent experiments. Stimulated T cells are indicated in black and unstimulated T cells in grey. Dot blots show single live CD14^−^CD19^−^CD3^+^CD4^+^ T cells. The frequency of all CD3^+^CD4^+^ T cell subsets identified upon stimulation are indicated in the corners of each plot.

The Rv2034 responding CD4^+^ T-cell clone was subsequently analyzed for Th17, Th2, regulatory and cytotoxicity T cell (CTL) markers to further specify its functional activities. The Rv2034 p81–100 peptide-induced CD154 expression correlated with IFN-γ expression (Figure 4A) while no IL-17 expression was observed. Transcription factor analysis revealed expression of T-bet (Figure 4B), a transcription factor that regulates Th1 development and controls IFN-γ production [42], [43], but there was no expression of GATA-3 (Th2 [44]), RORγt (Th17 [45], [46]) or FOXP3 (Treg [47]). T-bet was constitutively expressed in both p81–100 peptide stimulated and unstimulated T cells. Furthermore, the clone did not reveal any specific combination(s) of other reported T-cell regulatory markers (Figure 4C): no IL-10 could be detected although CD25 and CD39 [48] were constitutively expressed, which, however, are also expressed by activated non-Tregs [49], [50]. Of note, CD25 expressing non-Treg cells mostly express intermediate levels of CD25 (CD25^int^), whereas Tregs typically express high levels of CD25 (CD25^hi^) [49]. The expansion of our T-cell clone was mediated by IL-2 which theoretically could have enhanced CD25 expression [51]. Finally, the T-cell clone expressed granzyme B and CD107a but no detectable perforin (Figure 4D). The expression of granzyme B and CD107a was antigen-stimulation dependent. Cytotoxic CD4^+^ T cells have been reported not only in viral but also in mycobacterial infections [52]–[54]. The expression of the degranulation marker CD107a indicates the release of lysosomal products from granules after antigen-specific activation of the T cell, which likely includes granzyme B. Anti-CD107a antibodies were administered during antigen stimulation of the T-cell clone, because once CD107a is released with its vesicles’ granule contents and becomes integrated in the membrane of the cell, the antibodies will bind to CD107a and are subsequently internalized together with CD107, enabling detection of degranulation. Further studies are required to determine whether granulysin and other granzymes are released by the T-cell clone. These data were obtained by ICS using brefeldin A to accumulate intracellular cytokines by preventing their secretion. All subset conditions were also analyzed using monensin, which acts as protein transport inhibitor via disruption of trans-Golgi protein transport whereas BFA inhibits protein transport between endoplasmic reticulum (ER) and the Golgi apparatus. Monensin, however in our hands did not enhance any of the responses (data not shown). Taken together, these observations show that the Rv2034-specific CD4^+^ T cell-clone is a pure Th1 clone expressing IFN-γ, TNF-α, IL-2 and several cytotoxic granule markers.

### Determination of Genetic HLA-restriction of CD4^+^ T-cell Clone

**Figure 5:**
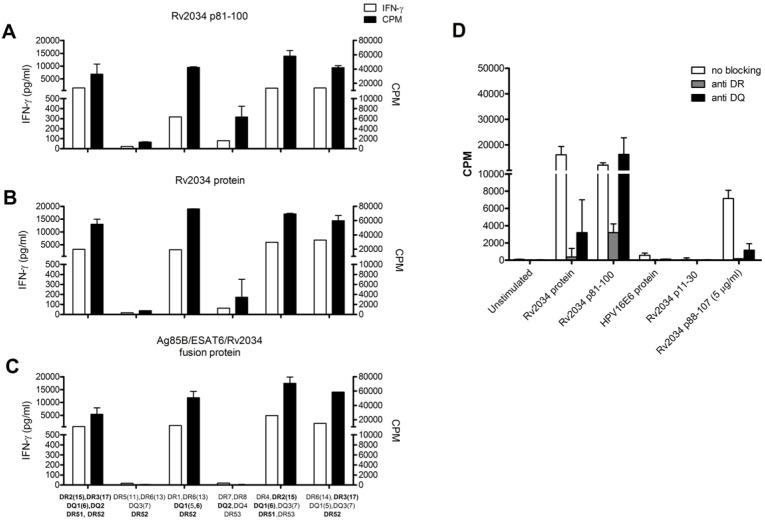
Restriction of Rv2034 p81–100 and p88–107 presentation by HLA-DR and -DQ molecules. To determine the HLA-DR and -DQ restriction of Rv2034 p81–100 responding CD4^+^ T-cell clone, T cells were incubated with Rv2034 p81–100 (A), Rv2034 protein (B) or Ag85B/ESAT-6/Rv2034 fusion protein (C), in the presence of irradiated PBMC with (mis)matched HLA-DR and/or DQ molecules. Both IFN-γ production (white bars) and proliferation (CPM) (black bars) was determined. CPM bars represent median ranging the highest and lowest value (n = 3). Matching HLA alleles are indicated in bold on the *x*-axis. HLA-DR and HLA-DQ molecules of APCs pulsed with either Rv2034 protein, Rv2034 p81–100, Rv2034 p88–107 (5 µg/ml) or control conditions, were blocked using monoclonal antibodies directed against HLA-DR or -DQ [21], and T-cell proliferation was analyzed (CPM) (D). Data is representative for three independent experiments.

To identify the genetic HLA-restriction of T-cell recognition of Rv2034 p81–100, the T-cell clone was cultured with (irradiated) PBMC from donors expressing various matching or mismatching HLA class II alleles in the presence of Rv2034 p81–100 (Figure 5A), Rv2034 protein (Figure 5B) and Ag85B/ESAT-6/Rv2034 fusion protein (Figure 5C). The autologous HLA genotype was HLA-DR2(15),-DR3(17),-DQ1(6),-DQ2,-DR51,-DR52. As expected, completely HLA-DRB1 and HLA-DQ mismatched PBMC (HLA-DR11,13 and HLA-DQ3(7)) failed to present peptide or protein antigens to the T-cell clone, even though these cells were matched for HLA-DRB3 (DR52), indicating that DR52 was not involved in Rv2034 p81–100 presentation. Both HLA-DR2(15)^+^/DQ1(6)^+^ and HLA-DR3(17)^+^/DQ1(5)^+^ PBMC induced strong T-cell activation. Since HLA-DR2(15) is in strong genetic linkage disequilibrium with HLA-DQ1(6) it was impossible to match for HLA-DR2(15) while mismatching for HLA-DQ1(6). HLA-DQ1(5,6)^+^ PBMC, which were mismatched for HLA-DR3(17) and HLA-DR2(15), also induced T-cell proliferation and IFN-γ in response to Rv2034 (fusion) protein and p81–100, whereas HLA-DQ2^+^ PBMC failed to present antigen. While the results were suggestive of HLA-DR3 and HLA-DQ1 presentation, the addition of purified monoclonal antibodies specific for only HLA-DR but not HLA-DQ backbone determinants resulted in a strong reduction of autologous APC presented Rv2034 p81–100 induced T-cell proliferation (Figure 5D). Thus the autologous responses towards Rv2034 p81–100 is predominantly HLA-DR and not HLA-DQ-restricted.

Surprisingly, however, while responses to the Rv2034 peptide p81–100 were HLA-DR restricted, the response to the whole Rv2034 protein was inhibited both by HLA-DR and by HLA-DQ-blocking antibodies (Figure 5D). Thus, while the epitope contained with p81–100 is HLA-DR restricted, the Rv2034 protein also contains an HLA-DQ1(6) restricted epitope sequence that can be recognized by the same T-cell clone. Of note, variable recognition was observed for Rv2034 p88–107 by the T-cell clone (Figure 4A and data not shown). Strong responses to p81–100 were observed at a concentration of 1 µg/ml (Figure 5D), whereas no responses to p88–107 could be detected at this concentration (data not shown). However, at a concentration of 5 µg/ml, p88–107 was able to activate the T-cell clone and, importantly, this could be blocked by both HLA-DR and HLA-DQ antibodies (Figure 5D). Thus, the Rv2034 p81–107 sequence possesses HLA-DR and HLA-DQ binding epitopes that were recognized by a single, apparently promiscuous CD4^+^ T-cell clone.

*In silico* HLA class II peptide binding predictions (SYFPEITHI [55], NetMHCIIpan 2.1, NetMHCII 2.2 and IEDB) revealed that Rv2034 p81–100 (LRTDLDRFWTRALTGYAQLI) indeed contains sequences with high predicted binding affinity for multiple HLA-DRB1 and HLA-DQ molecules (Table S1). HLA-DR2(15) and HLA-DR3(17) show binding potential for LDRFWTRAL and TDLDRFWTR, which are located at the beginning of the peptide while the TRALTGYAQ sequence has predicted potential to bind to HLA-DQ1(6). Although Rv2034 p88–107 (FWTRALTGYAQLIDSEGDDT) contains a potential epitope (ALTGYAQLI) that could bind to HLA-DR2(15), it contains limited predicted epitopes available for HLA-DR3(17). Nevertheless, predicted HLA-DQ1(6) epitopes were identified (TRALTGYAQ and TGYAQLIDS) (Table S2). As a control, very few predicted epitopes were observed for Rv2034 p11–30 (Table S3), indicating that the p81–107 sequence has a rather unique high (predicted) epitope density. These data are best explained by a model in which the T-cell clone recognizes Rv2034 p81–100 and Rv2034 p88–107 via multiple HLA molecules, possibly including HLA-DR2, -DR3 as well as HLA-DQ1 registers, but that the dominant response to the protein is HLA-DR restricted. Such promiscuous peptides, capable of being presented by multiple HLA molecules, have been shown for several mycobacterial proteins [56], including HspX (16 kDa protein) [57], TB10.4 [58] and Hsp65 [59]. In addition, some (*Mtb* antigen) studies showed possible presentation of a single epitope, specific for one clone, via both HLA-DR and HLA-DQ [57], [60]. Other studies also demonstrated promiscuity of viral epitope presentation [61], [62]. The presentation of a specific epitope via multiple HLA molecules has been described as HLA cross-restriction [63], [64] and this promiscuity phenomenon is suggested to be extensively present [65], [66]. Promiscuous *Mtb* peptides are particularly interesting for development of novel *Mtb* vaccines since they would be able to cover many different HLA alleles throughout different populations.

### Recognition of Rv2034 in *Mtb* Lysate

**Figure 6:**
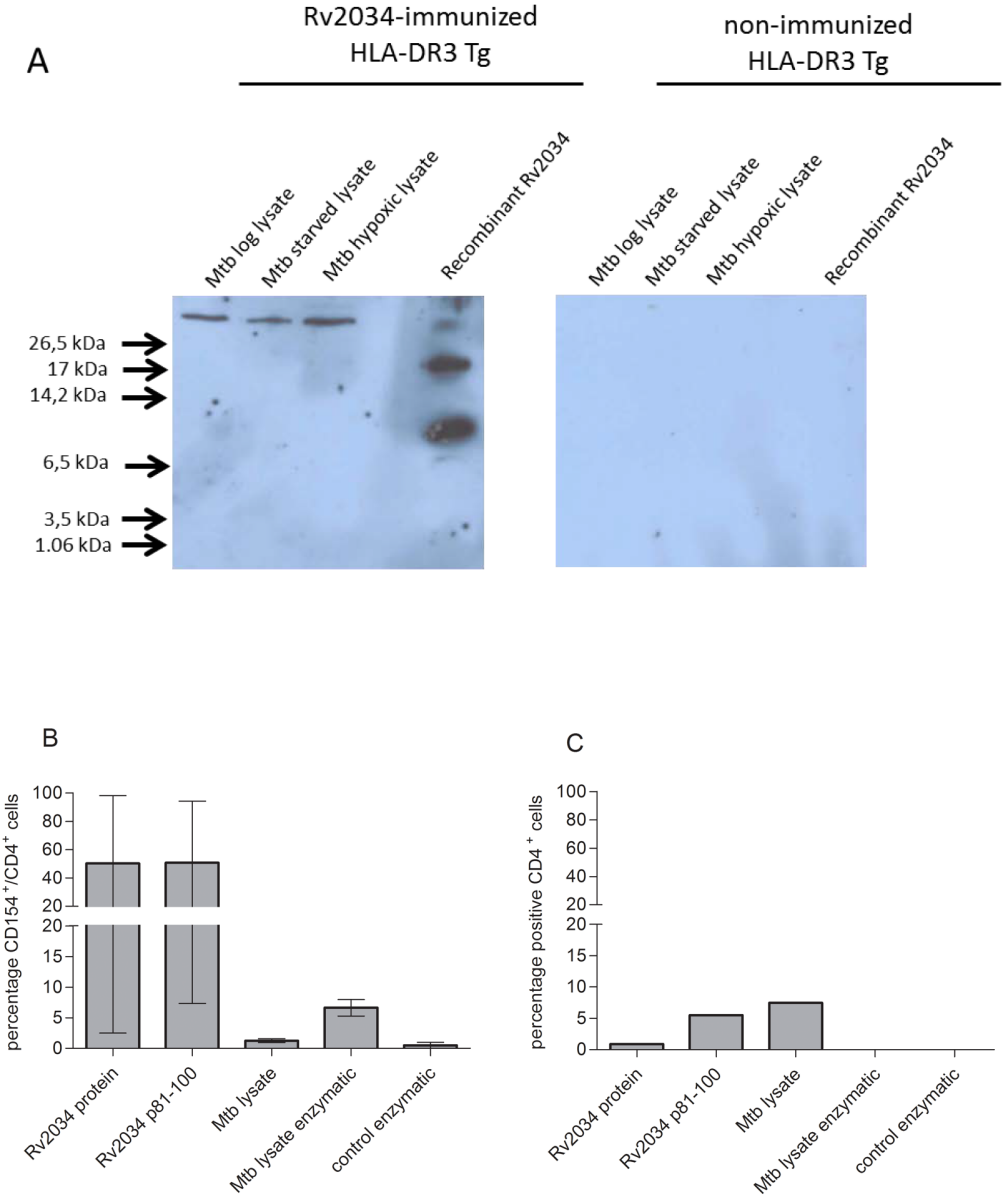
Recognition of native Rv2034 in *Mtb* lysate by CD4^+^ T-cell clone. *Mtb* lysates (10 µg) derived from *Mtb* grown in log-phase-, starvation- and hypoxic- conditions and recombinant protein Rv2034 (0.4 µg) were analyzed by western blotting for their recognition by specific antibodies using sera of Rv2034-immunized or non-immunized HLA-DR3 transgenic mice (A). Monocyte derived matured DCs were loaded with Rv2034 p81–100, Rv2034 protein and different conditions of *Mtb* lysate (100 µg/ml) for 24h. Subsequently, the CD4^+^ T-cell clone was incubated with these antigen-loaded DCs and after 2 hours BFA was added and incubated o/n. CD154 expression was determined using flow cytometry (B). Data represent two independent experiments. An HA-specific T-cell clone was included as a negative control (C). Medium background values were subtracted for each response.

We next wished to demonstrate recognition of native *Mtb* expressed Rv2034 protein by the T-cell clone in order to better understand its possible role during *Mtb* infection. First we examined whether Rv2034 protein was expressed in *Mtb* lysates by western blot analysis. Using sera of Rv2034-immunized (HLA-DR3 transgenic) mice, expression of Rv2034 was identified in both log phase *Mtb* lysate and *Mtb* grown during hypoxic and starvation conditions (Figure 6A). Although the protein size was predicted to be approximately 12 kDa (http://www.sciencegateway.org/tools/proteinmw.htm), the native Rv2034 present in the lysates was larger than expected. The recombinant Rv2034 protein includes a histidine tag and thus was expected to have a molecular weight of ∼14 kDa. Since Rv2034 contains dimerization sites [67], this higher relative molecular mass could be due to formation of multimers. Alternatively but not mutually exclusive, post-translational modifications or binding to other molecules might be responsible for the higher molecular weight. Two bands were clearly visible, the smallest band likely relating to Rv2034 protein monomer, whereas the upper band probably indicates multimer formation. The binding of antibodies present in the sera of Rv2034-immunized mice was specific since no bands were observed using sera from non-immunized HLA-DR3 mice. Indeed, Rv2034 protein was also previously identified in *in vitro* grown *Mtb* cultures [10], [68], [69], specifically in lipid (membrane) associated fractions, indicating that the protein is indeed expressed by *Mtb*.

We next analyzed the T-cell clone’s reactivity against *Mtb* lysate to see if the T-cell clone was able to detect the native Rv2034 protein. HLA-matched matured dendritic cells (DC) were used as antigen-presenting cells and activated CD4^+^CD154^+^ responding T cells analyzed (Figure 6B). Both Rv2034 protein and Rv2034 p81–100 strongly activated the T-cell clone, whereas no activation was observed in response to *Mtb* lysate. The control T-cell clone, specific for an influenza haemagglutinin (HA) epitope p307–319 [62], [70], showed low level non-specific activation upon lysate exposure (Figure 6C). Rv2034 might be difficult for DC to process and present due to possible posttranslational modifications, dimer formation [67], or associations to other molecules (Rv2034 is lipid or membrane associated [68], [69]). It should be noted, however, that Rv2034 is efficiently recognized by T cells from tuberculin skin test-positive, *in vitro* ESAT-6/CFP10-responsive individuals in whole blood or PBMC assays [10]. To improve processing of the lysate by the APC, the lysate was further digested and homogenized using proteinase K (serine protease) and lysozyme (peptidoglycan degradation) enzymes. Treating the *Mtb* lysate concentration resulted in an increased population of activated CD4^+^ T cells. Importantly, incubating the cells with a control sample containing the same amount of inactivated enzymes as used for the lysate did not show any recognition. Of note, the above mentioned non-specific activation of the HA specific T-cell clone was lost upon enzyme treatment of the lysate. This further indicates that the observed T-cell activation is *Mtb* lysate-specific.

Nonetheless, activation of the T-cell clone using the enzyme treated *Mtb* lysate did not reach the full level of activation as observed for recombinant Rv2034 protein and Rv2034 p81–100. Obviously, *Mtb* lysate is a very crude product containing many different proteins as well as other molecules and fragments, such that only low levels of Rv2034 might be present in the lysate. Furthermore, only activation was observed after enzyme treatment verifying that Rv2034 was associated to certain structures that prevented or hindered correct processing by the matured DC. A lysate concentration dependent effect on T cells has been observed previously [52].

**Figure 7:**
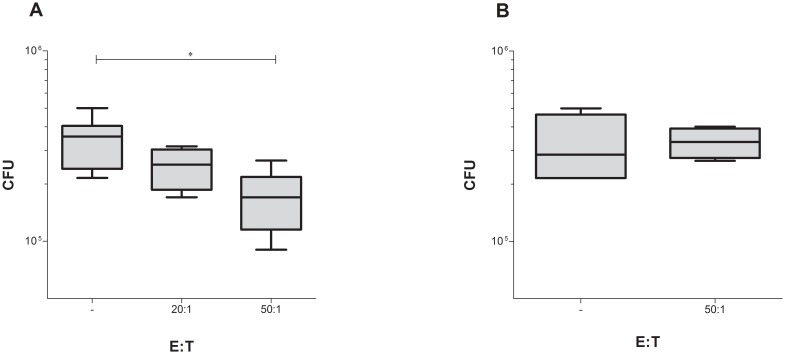
CD4^+^ T-cell clone has *Mtb* inhibitory properties. Autologous PBMC were loaded with *Mtb* (10 MOI) and the Rv2034 T-cell clone was added at an effector/target (E/T) ratio of 20∶1 and 50∶1 as indicated on the *x*-axis. CFU were determined after o/n incubation. Eight replicates from two independent experiments were included for 50∶1 and only target cells condition, four replicates were included for 20∶1 condition (A). The HA-specific clone was included as a negative control (n = 4) (B). The horizontal line indicates the median value and the outer boundaries of the box represents the 25^th^ and 75^th^ percentiles. The whiskers indicate the highest and lowest values. A Mann-Whitney *U* test was performed to analyze the difference between CFU. A *p*-value≤0.05 was considered significant and indicated with an asterisk.

The T-cell clone thus expressed cytotoxic markers (Figure 4) and recognized native Rv2034 in *Mtb* lysate (Figure 6B). To test whether the T-cell clone could directly inhibit *Mtb* outgrowth from infected APCs, autologous monocytes were infected with *Mtb* and the T-cell clone was added in an effector/target ratio of 20∶1 and 50∶1. Interestingly, addition of the T-cell clone resulted in a significant decrease of CFU in a dose-dependent fashion (*p* = 0.0038, Figure 7A), whereas this was not observed when the HA-specific T-cell clone was added to the *Mtb* loaded APCs (Figure 7B). Thus the T-cell clone is able to inhibit the outgrowth of *Mtb* from infected cells directly.

### Concluding Remarks

A better understanding of T-cell immunity during infection and following vaccination is important in many diseases, including tuberculosis. Here, we describe an approach that can be used for the rapid generation of antigen specific T-cell clones irrespective of functional properties such as cytokine secretion, in order to study antigen specific T-cell subsets in more detail, even for low frequency responses. Both CD4^+^, CD8^+^ and DN T-cell clones could be generated, which showed responses to the secreted TB10.4 or the IVE-TB Rv2034 *Mtb* antigens. An Rv2034 specific CD4^+^ T-cell clone was further analyzed, its antigen-specificity confirmed for (native) Rv2034 protein, a dominant peptide epitope identified and a promiscuous HLA-DR/DQ restriction pattern elucidated. Furthermore, the T-cell clone expressed Th1 and cytotoxicity related markers, and had significant *Mtb* inhibition activity. Using this approach, the T-cell responses to different *Mtb* specific antigens, including phase-dependent and IVE-TB antigens, can be further analyzed which should help to understand the immune response to *Mtb*. In addition, screening of T-cell clones based on marker expression and functionality reveals T-cell subsets that could play a role in the host defence to TB, and our method allows the analysis of such T cell subsets in a broader population studies such as TB patients.

## Supporting Information

Figure S1 **MACS sort of CD154 positive T cells from Rv2034 peptide pool stimulated PBMC.** PBMC were stimulated for 16 hours and labeled with anti-CD154 PE and anti-PE microbeads and sorted. A small sample was collected to analyze the positive CD154 positive population. Subsequently after sorting CD154 negative and CD154 positive collections were stained as well.
(PDF)

Table S1 **HLA-DR and -DQ binding predictions for Rv2034 p81–100 (LRTDLDRFWTRALTGYAQLI).**
(PDF)

Table S2 **HLA-DR and -DQ binding predictions for Rv2034 p88–107 (FWTRALTGYAQLIDSEGDDT).**
(PDF)

Table S3 **HLA-DR and -DQ binding predictions for Rv2034 p11–30 (WQALADGTRRAIVERLAHGP).**
(PDF)
