# Supplementary figures and images for: Clonal Analysis of the T-Cell Response to In Vivo Expressed Mycobacterium tuberculosis Protein Rv2034, Using a CD154 Expression Based T-Cell Cloning Method
Source: PLoS One. 2014 Jun 6;9(6):e99203. doi: 10.1371/journal.pone.0099203 (PMC4048274; doi:10.1371/journal.pone.0099203)

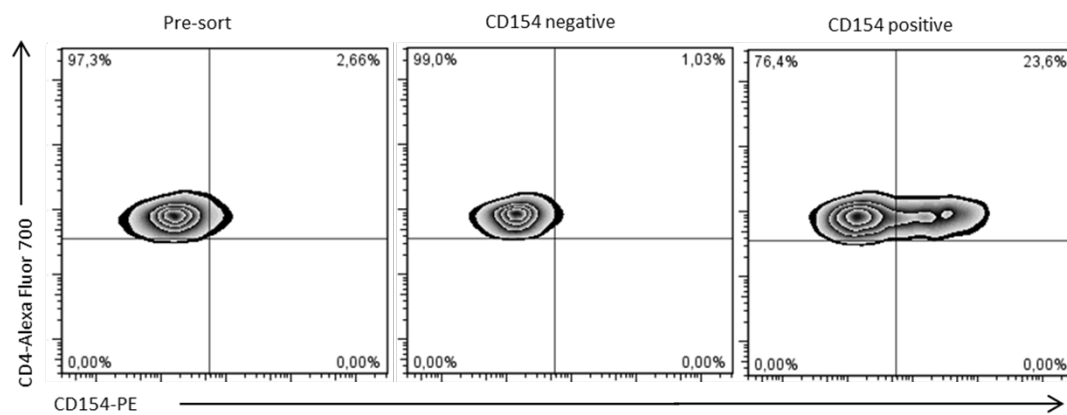

Supplement: Figure S1 — MACS sort of CD154 positive T cells from Rv2034 peptide pool stimulated PBMC. PBMC were stimulated for 16 hours and labeled with anti-CD154 PE and anti-PE microbeads and sorted. A small sample was collected to analyze the positive CD154 positive population. Subsequently after sorting CD154 negative and CD154 positive collections were stained as well. (PDF) [file pone.0099203.s001.pdf]
