# Supplementary material for: Clonal Analysis of the T-Cell Response to In Vivo Expressed Mycobacterium tuberculosis Protein Rv2034, Using a CD154 Expression Based T-Cell Cloning Method
Source: PLoS One. 2014 Jun 6;9(6):e99203. doi: 10.1371/journal.pone.0099203 (PMC4048274; doi:10.1371/journal.pone.0099203)
Supplement: Table S1 — HLA-DR and -DQ binding predictions for Rv2034 p81–100 (LRTDLDRFWTRALTGYAQLI). (PDF) [file pone.0099203.s002.pdf]

Table S1

Table S1. HLA-DR and -DQ binding predictions for Rv2034 p81-100 (LRTDLDRFWTRALTGYAQLI)

**HLA-DR**	**Allele molecule**	**SYFPEITHI [a]**	**NetMHCIIpan 2.1**	**NetMHCII 2.2**	**IEDB**	**IEDB**
**Sequence**	**HLA-DRB1**	**Score**	**Affinity nM**	**Affinity nM**	**arbpython (ARB)<br>Affinity nM**	**SMM<br>Affinity nM**
DRFWTRALTGYAQLI	0101	**25**	**9.71 (SB)<br>WTRALTGYA**	**18.2 (SB)<br>TRALTGYAQ**	**21.7 (SB)<br>WTRALTGYA**	**148.0 (WB)<br>TRALTGYAQ**
RTDLDRFWTRALTGY	0101	23	**308.33 (WB)<br>LDRFWTRAL**	**97.8 (WB)<br>LDRFWTRAL**	**422.5 (WB)<br>FWTRALTGY**	**465.0 (WB)<br>LDRFWTRAL**
RTDLDRFWTRALTGY	0301 (17)	19	1562.32<br>TDLDRFWTR	523.4<br>TDLDRFWTR	**397.1 (WB)<br>TDLDRFWTR**	5181.0<br>TDLDRFWTR
LRTDLDRFWTRALTG	0301 (17)	2	893.68<br>TDLDRFWTR	**208.0 (WB)<br>TDLDRFWTR**	**189.8 (WB)<br>LRTDLDRFW**	1164.0<br>TDLDRFWTR
RTDLDRFWTRALTGY	0401	**26**	1230.41<br>LDRFWTRAL	510.2<br>RFWTRALTG	1045.4<br>LDRFWTRAL	824.0<br>LDRFWTRAL
DRFWTRALTGYAQLI	0401	**28**	**75.81 (WB)<br>WTRALTGYA**	**216.0 (WB)<br>WTRALTGYA**	1500.4<br>WTRALTGYA	1294.0<br>TRALTGYAQ
DLDRFWTRALTGYAQ	0401	**0**	**254.70 (WB)<br>WTRALTGYA**	**208.7 (WB)<br>FWTRALTGY**	1045.4<br>LDRFWTRAL	1022.0<br>LDRFWTRAL
RTDLDRFWTRALTGY	0701	24	**236.64 (WB)<br>LDRFWTRAL**	**181.2 (WB)<br>RFWTRALTG**	**129.0 (WB)<br>LDRFWTRAL**	**490.0 (WB)<br>LDRFWTRAL**
DRFWTRALTGYAQLI	0701	**28**	**56.19 (WB)<br>WTRALTGYA**	**221.4 (WB)<br>RFWTRALTG**	1364.7<br>RFWTRALTG	1052.0<br>WTRALTGYA
LRTDLDRFWTRALTG	0701	**4**	**396.16 (WB)<br>LDRFWTRAL**	**169.1 (WB)<br>RFWTRALTG**	**129.0 (WB)<br>LDRFWTRAL**	503.0<br>LDRFWTRAL
DRFWTRALTGYAQLI	0801	ND	**45.80 (SB)<br>WTRALTGYA**	ND	ND	ND
DRFWTRALTGYAQLI	0901	ND	**19.99 (SB)<br>WTRALTGYA**	**21.2 (SB)<br>WTRALTGYA**	1575.8<br>WTRALTGYA	**176.0 (WB)<br>WTRALTGYA**
DRFWTRALTGYAQLI	1001	ND	**36.40 (SB)<br>WTRALTGYA**	ND	ND	ND
DRFWTRALTGYAQLI	1101	18	**47.00 (SB)<br>WTRALTGYA**	**73.9 (WB)<br>WTRALTGYA**	506.2<br>WTRALTGYA	793.0<br>WTRALTGYA
DLDRFWTRALTGYAQ	1101	7	**118.48 (WB)<br>WTRALTGYA**	**44.3 (SB)<br>DRFWTRALT**	506.2<br>WTRALTGYA	542.0<br>LDRFWTRAL
TDLDRFWTRALTGYA	1101	9	**176.31 (WB)<br>WTRALTGYA**	**47.9 (SB)<br>DRFWTRALT**	506.2<br>WTRALTGYA	**264.0 (WB)<br>LDRFWTRAL**
DRFWTRALTGYAQLI	1201	ND	**30.49 (SB)<br>WTRALTGYA**	ND	**24.7 (SB)<br>DRFWTRALT**	584.0<br>FWTRALTGY
TDLDRFWTRALTGYA	1201	ND	**54.33 (WB)<br>LDRFWTRAL**	ND	**24.7 (SB)<br>DRFWTRALT**	598.0<br>FWTRALTGY
RTDLDRFWTRALTGY	1301	ND	896.73<br>LDRFWTRAL	ND	ND	ND
LDRFWTRALTGYAQL	1401	ND	518.20<br>WTRALTGYA	ND	ND	ND
RTDLDRFWTRALTGY	1501	**28**	**160.58 (WB)<br>LDRFWTRAL**	**81.6 (WB)<br>LDRFWTRAL**	**260.8 (WB)<br>LDRFWTRAL**	**292.0 (WB)<br>LDRFWTRAL**
DRFWTRALTGYAQLI	1501	8	**144.92 (WB)<br>WTRALTGYA**	**292.7 (WB)<br>WTRALTGYA**	1164.6 ALTGYAQLI	1208.0<br>WTRALTGYA
DRFWTRALTGYAQLI	1601	ND	**88.90 (WB)<br>WTRALTGYA**	ND	ND	ND

**HLA-DQ**	**Allele molecule**	**SYFPEITHI [a]**	**NetMHCIIpan 2.1**	**NetMHCII 2.2**	**IEDB**	**IEDB**
**Sequence**	**HLA-DQA1-DQB1**	**Score**	**Affinity nM**	**Affinity nM**	**arbpython (ARB)<br>Affinity nM**	**SMM<br>Affinity nM**
RTDLDRFWTRALTGY	0101-0501 (DQ1(5))	ND	ND	3350.1<br>LDRFWTRAL	17544.9<br>LDRFWTRAL	2122.0<br>LDRFWTRAL
LDRFWTRALTGYAQL	0101-0501 (DQ1(5))	ND	ND	3624.5<br>LDRFWTRAL	6495.7<br>RALTGYAQL	1964.0<br>LDRFWTRAL
LDRFWTRALTGYAQL	0102-0602 (DQ1(6))	ND	ND	**110.3 (WB)<br>TRALTGYAQ**	**416.4 (WB)<br>TRALTGYAQ**	863.0<br>TRALTGYAQ
DRFWTRALTGYAQLI	0301-0302 (DQ3(8))	ND	ND	741.7<br>TRALTGYAQ	1508.9<br>RALTGYAQL	2599.0<br>TRALTGYAQ
DRFWTRALTGYAQLI	0401-0402 (DQ4)	ND	ND	2289.2<br>ALTGYAQLI	8839.3<br>TRALTGYAQ	5501.0<br>TRALTGYAQ
DRFWTRALTGYAQLI	0501-0201 (DQ2)	ND	ND	2258.9<br>ALTGYAQLI	3126.6<br>TRALTGYAQ	4043.0<br>RALTGYAQL
DRFWTRALTGYAQLI	0501-0301 (DQ3(7))	ND	ND	**39.8 (SB)<br>ALTGYAQLI**	**268.7 (WB)<br>RALTGYAQL**	**150.0 (WB)<br>TRALTGYAQ**

[a] SYFPEITHI scores ≥ 25 are considered positive

Affinity nM: Strong binder (SB) = between 1 and 50 nM
Weak binder (WB) = between 50 and 500 nM

Core amino acids sequences are indicated along with Affinity nM values
