# Supplementary material for: Clonal Analysis of the T-Cell Response to In Vivo Expressed Mycobacterium tuberculosis Protein Rv2034, Using a CD154 Expression Based T-Cell Cloning Method
Source: PLoS One. 2014 Jun 6;9(6):e99203. doi: 10.1371/journal.pone.0099203 (PMC4048274; doi:10.1371/journal.pone.0099203)
Supplement: Table S2 — HLA-DR and -DQ binding predictions for Rv2034 p88–107 (FWTRALTGYAQLIDSEGDDT). (PDF) [file pone.0099203.s003.pdf]

Table S2

Table S2. HLA-DR and -DQ binding predictions for Rv2034 p88-107 (FWTRALTGYAQLIDSEGDDT)

HLA-DR	Allele molecule	SYFPEITHI	NetMHCIIpan 2.1	NetMHCII 2.2	IEDB	IEDB
**Sequence**	**HLA-DRB1**	**Score**	**Affinity nM**	**Affinity nM**	**arbpython (ARB) Affinity nM**	**SMM Affinity nM**
LTGYAQLIDSEGDDT	0101	18	**312.24 (WB) YAQLIDSEG**	**461.4 (WB) YAQLIDSEG**	**231.3 (WB) YAQLIDSEG**	**253.0 (WB) YAQLIDSEG**
TRALTGYAQLIDSEG	0101	9	**181.26 (WB) ALTGYAQLI**	**92.6 (WB) ALTGYAQLI**	**24.6 (SB) ALTGYAQLI**	**160.0 (WB) ALTGYAQLI**
FWTRALTGYAQLIDS	0101	10	**19.73 (SB) WTRALTGYA**	**35.8 (SB) TRALTGYAQ**	**21.7 (SB) WTRALTGYA**	**199.0 (WB) TRALTGYAQ**
TRALTGYAQLIDSEG	0301 (17)	18	8678.79 ALTGYAQLI	8615.4 LTGYAQLID	6774.6 LTGYAQLID	12827.0 ALTGYAQLI
WTRALTGYAQLIDSE	0301 (17)	4	7642.93 ALTGYAQLI	7257.2 LTGYAQLID	6774.6 LTGYAQLID	12186.0 ALTGYAQLI
ALTGYAQLIDSEGDD	0301 (17)	1	10489.46 GYAQLIDSE	12849.8 LTGYAQLID	**134.5 (WB) QLIDSEGDD**	11880.0 ALTGYAQLI
LTGYAQLIDSEGDDT	0401	16	845.88 YAQLIDSEG	**298.6 (WB) YAQLIDSEG**	1206.2 YAQLIDSEG	910.0 YAQLIDSEG
FWTRALTGYAQLIDS	0401	6	**235.93 (WB) WTRALTGYA**	623.0 TRALTGYAQ	1500.4 WTRALTGYA	1752.0 TRALTGYAQ
FWTRALTGYAQLIDS	0701	16	**149.85 (WB) WTRALTGYA**	1010.6 ALTGYAQLI	1564.2 FWTRALTGY	1435.0 ALTGYAQLI
FWTRALTGYAQLIDS	0801	ND	**114.75 (WB) WTRALTGYA**	ND	ND	ND
FWTRALTGYAQLIDS	0901	ND	**49.71 (SB) WTRALTGYA**	**44.5 (SB) WTRALTGYA**	1575.8 WTRALTGYA	508.0 WTRALTGYA
FWTRALTGYAQLIDS	1001	ND	**90.27 (WB) WTRALTGYA**	ND	ND	ND
LTGYAQLIDSEGDDT	1101	22	5316.28 YAQLIDSEG	4891.0 YAQLIDSEG	10335.7 YAQLIDSEG	2749.0 YAQLIDSEG
FWTRALTGYAQLIDS	1101	0	**129.36 (WB) WTRALTGYA**	**270.2 (WB) TRALTGYAQ**	506.2 WTRALTGYA	1727.0 TRALTGYAQ
WTRALTGYAQLIDSE	1201	ND	**130.28 (WB) WTRALTGYA**	ND	**76.9 (WB) GYAQLIDSE**	5793.0 ALTGYAQLI
FWTRALTGYAQLIDS	1201	ND	**73.92 (WB) WTRALTGYA**	ND	**226.0 (WB) FWTRALTGY**	1436.0 FWTRALTGY
FWTRALTGYAQLIDS	1301	ND	1301.10 ALTGYAQLI	ND	ND	ND
FWTRALTGYAQLIDS	1401	ND	1119.26 WTRALTGYA	ND	ND	ND
TRALTGYAQLIDSEG	1501	**26**	1920.39 ALTGYAQLI	2419.7 ALTGYAQLI	1164.6 ALTGYAQLI	2869.0 ALTGYAQLI
LTGYAQLIDSEGDDT	1501	4	6484.58 YAQLIDSEG	7948.3 LTGYAQLID	499550.7 LIDSEGDDT	8760.0 LTGYAQLID
FWTRALTGYAQLIDS	1501	4	**423.74 (WB) WTRALTGYA**	**404.7 (WB) WTRALTGYA**	1164.6 ALTGYAQLI	1725.0 TRALTGYAQ
FWTRALTGYAQLIDS	1601	ND	**244.06 (WB) WTRALTGYA**	ND	ND	ND

HLA-DQ	Allele molecule	SYFPEITHI	NetMHCIIpan 2.1	NetMHCII 2.2	IEDB	IEDB
**Sequence**	**HLA-DQA1-DQB1**	**Score**	**Affinity nM**	**Affinity nM**	**arbpython (ARB) Affinity nM**	**SMM Affinity nM**
WTRALTGYAQLIDSE	0101-0501 (DQ1(5))	ND	ND	932.5 ALTGYAQLI	6495.7 RALTGYAQL	3032.0 LTGYAQLID
FWTRALTGYAQLIDS	0102-0602 (DQ1(6))	ND	ND	**200.7 (WB) TRALTGYAQ**	**416.4 (WB) TRALTGYAQ**	875.0 TRALTGYAQ
WTRALTGYAQLIDSE	0102-0602 (DQ1(6))	ND	ND	**278.2 (WB) TGYAQLIDS**	**416.4 (WB) TRALTGYAQ**	982.0 TGYAQLIDS
WTRALTGYAQLIDSE	0301-0302 (DQ3(8))	ND	ND	791.3 TRALTGYAQ	1508.9 RALTGYAQL	2218.0 ALTGYAQLI
RALTGYAQLIDSEGD	0301-0302 (DQ3(8))	ND	ND	918.5 GYAQLIDSE	1508.9 RALTGYAQL	2038.0 GYAQLIDSE
WTRALTGYAQLIDSE	0401-0402 (DQ4)	ND	ND	720.1 LTGYAQLID	8839.3 TRALTGYAQ	1907.0 LTGYAQLID
TRALTGYAQLIDSEG	0401-0402 (DQ4)	ND	ND	839.6 GYAQLIDSE	6060.8 YAQLIDSEG	1823.0 GYAQLIDSE
LTGYAQLIDSEGDDT	0501-0201 (DQ2)	ND	ND	576.6 GYAQLIDSE	2693.9 GYAQLIDSE	**498.0 (WB) GYAQLIDSE**
WTRALTGYAQLIDSE	0501-0201 (DQ2)	ND	ND	**383.2 (WB) GYAQLIDSE**	2693.9 GYAQLIDSE	1259.0 RALTGYAQL
FWTRALTGYAQLIDS	0501-0301 (DQ3(7))	ND	ND	**31.6 (SB) TGYAQLIDS**	**268.7 (WB) RALTGYAQL**	**116.0 (WB) ALTGYAQLI**

[a] SYFPEITHI scores ≥ 25 are considered positive

Affinity nM: Strong binder (SB) = between 1 and 50 nM
Weak binder (WB) = between 50 and 500 nM

Core amino acids sequences are indicated along with Affinity nM values
