# Supplementary material for: Clonal Analysis of the T-Cell Response to In Vivo Expressed Mycobacterium tuberculosis Protein Rv2034, Using a CD154 Expression Based T-Cell Cloning Method
Source: PLoS One. 2014 Jun 6;9(6):e99203. doi: 10.1371/journal.pone.0099203 (PMC4048274; doi:10.1371/journal.pone.0099203)
Supplement: Table S3 — HLA-DR and -DQ binding predictions for Rv2034 p11–30 (WQALADGTRRAIVERLAHGP). (PDF) [file pone.0099203.s004.pdf]

Table S3

Table S3. HLA-DR and -DQ binding predictions for Rv2034 p11-30 (WQALADGTRRAIVERLAHGP)

**HLA-DR**	**Allele molecule**	**SYFPEITHI**	**NetMHCIIpan 2.1**	**NetMHCII 2.2**	**IEDB**	**IEDB**
**Sequence**	**HLA-DRB1**	**Score**	**Affinity nM**	**Affinity nM**	**arbpython (ARB) Affinity nM**	**SMM Affinity nM**
WQALADGTRRAIVER	0101	17	936.57 WQALADGTR	1338.2 WQALADGTR	**328.2 (WB) WQALADGTR**	1280.0 WQALADGTR
ADGTRRAIVERLAHG	0101	10	548.27 TRRAIVERL	**217.2 (WB) TRRAIVERL**	**162.3 (WB) TRRAIVERL**	966.0 TRRAIVERL
DGTRRAIVERLAHGP	0101	17	649.35 TRRAIVERL	**358.4 (WB) TRRAIVERL**	**162.3 (WB) TRRAIVERL**	704.0 RRAIVERLA
WQALADGTRRAIVER	0301 (17)	17	**382.05(WB) ALADGTRRA**	3657.0 ALADGTRRA	778.5 WQALADGTR	1964.0 ALADGTRRA
LADGTRRAIVERLAH	0301 (17)	2	2573.34 TRRAIVERL	2246.2 TRRAIVERL	7041.1 RRAIVERLA	3947.0 TRRAIVERL
WQALADGTRRAIVER	0401	20	2619.02 ALADGTRRA	1776.5 ALADGTRRA	6144.3 ALADGTRRA	1671.0 ALADGTRRA
QALADGTRRAIVERL	0401	12	3992.11 ALADGTRRA	1705.9 ALADGTRRA	1899.1 TRRAIVERL	2629.0 LADGTRRAI
WQALADGTRRAIVER	0701	16	2050.02 LADGTRRAI	7737.1 LADGTRRAI	7861.3 LADGTRRAI	2323.0 ADGTRRAIV
LADGTRRAIVERLAH	0701	0	1160.30 TRRAIVERL	2146.9 TRRAIVERL	**416.1 (WB) TRRAIVERL**	1726.0 TRRAIVERL
QALADGTRRAIVERL	0701	16	1391.66 TRRAIVERL	1753.3 TRRAIVERL	**416.1 (WB) TRRAIVERL**	1222.0 LADGTRRAI
DGTRRAIVERLAHGP	0801	ND	**102.49 (WB) RAIVERLAH**	ND	ND	ND
LADGTRRAIVERLAH	0901	ND	942.60 TRRAIVERL	1257.0 RRAIVERLA	7453.7 TRRAIVERL	796.0 RRAIVERLA
WQALADGTRRAIVER	0901	ND	1869.34 WQALADGTR	1622.3 LADGTRRAI	4085.1 WQALADGTR	2363.0 LADGTRRAI
ADGTRRAIVERLAHG	1001	ND	1264.06 TRRAIVERL	ND	ND	ND
DGTRRAIVERLAHGP	1101	16	**499.56 (WB) RAIVERLAH**	**90.7 (WB) RAIVERLAH**	**437.9 (WB) TRRAIVERL**	1172.0 RAIVERLAH
QALADGTRRAIVERL	1101	9	1500.09 LADGTRRAI	589.6 LADGTRRAI	**437.9 (WB) TRRAIVERL**	981.0 LADGTRRAI
DGTRRAIVERLAHGP	1201	ND	**209.21 (WB) RAIVERLAH**	ND	**233.7 (WB) AIVERLAHG**	5680.0 AIVERLAHG
ALADGTRRAIVERLA	1201	ND	**450.26 (WB) TRRAIVERL**	ND	**236.4 (WB) RRAIVERLA**	38230.0 ALADGTRRA
DGTRRAIVERLAHGP	1301	ND	1347.99 TRRAIVERL	ND	ND	ND
DGTRRAIVERLAHGP	1401	ND	1333.73 RAIVERLAH	ND	ND	ND
WQALADGTRRAIVER	1501	14	3684.32 ALADGTRRA	7965.6 ADGTRRAIV	39071.2 LADGTRRAI	8956.0 ALADGTRRA
DGTRRAIVERLAHGP	1501	4	2064.34 TRRAIVERL	3040.1 AIVERLAHG	**420.3 (WB) IVERLAHGP**	2426.0 RRAIVERLA
DGTRRAIVERLAHGP	1601	ND	1556.01 RRAIVERLA	ND	ND	ND

**HLA-DQ**	**Allele molecule**	**SYFPEITHI**	**NetMHCIIpan 2.1**	**NetMHCII 2.2**	**IEDB**	**IEDB**
**Sequence**	**HLA-DQA1-DQB1**	**Score**	**Affinity nM**	**Affinity nM**	**arbpython (ARB) Affinity nM**	**SMM Affinity nM**
ADGTRRAIVERLAHG	0101-0501 (DQ1(5))	ND	ND	20442.7 TRRAIVERL	39420.9 TRRAIVERL	19682.0 TRRAIVERL
WQALADGTRRAIVER	0101-0501 (DQ1(5))	ND	ND	30725.4 ADGTRRAIV	30620.6 LADGTRRAI	25918.0 WQALADGTR
ADGTRRAIVERLAHG	0102-0602 (DQ1(6))	ND	ND	**342.7 (WB) TRRAIVERL**	2666.1 TRRAIVERL	1450.0 RRAIVERLA
WQALADGTRRAIVER	0102-0602 (DQ1(6))	ND	ND	5621.9 ADGTRRAIV	1049.0 LADGTRRAI	3782.0 ADGTRRAIV
LADGTRRAIVERLAH	0301-0302 (DQ3(8))	ND	ND	1204.6 DGTRRAIVE	9616.6 RAIVERLAH	1551.0 TRRAIVERL
QALADGTRRAIVERL	0401-0402 (DQ4)	ND	ND	1364.5 DGTRRAIVE	10443.7 TRRAIVERL	2530.0 GTRRAIVER
LADGTRRAIVERLAH	0401-0402 (DQ4)	ND	ND	1758.0 DGTRRAIVE	10443.7 TRRAIVERL	2523.0 GTRRAIVER
DGTRRAIVERLAHGP	0501-0201 (DQ2)	ND	ND	1730.8 TRRAIVERL	5997.0 TRRAIVERL	3385.0 TRRAIVERL
QALADGTRRAIVERL	0501-0201 (DQ2)	ND	ND	**484.9 (WB) TRRAIVERL**	5997.0 TRRAIVERL	4564.0 GTRRAIVER
ALADGTRRAIVERLA	0501-0301 (DQ3(7))	ND	ND	**143.7 (WB) TRRAIVERL**	**14.6 (SB) ADGTRRAIV**	**255.0 (WB) ADGTRRAIV**
QALADGTRRAIVERL	0501-0301 (DQ3(7))	ND	ND	**154.4 (WB) ADGTRRAIV**	**14.6 (SB) ADGTRRAIV**	**237.0 (WB) ADGTRRAIV**

[a] SYFPEITHI scores ≥ 25 are considered positive

Affinity nM: Strong binder (SB) = between 1 and 50 nM
Weak binder (WB) = between 50 and 500 nM

Core amino acids sequences are indicated along with Affinity nM values
